# Oxidative Stress in the Pathogenesis of Aorta Diseases as a Source of Potential Biomarkers and Therapeutic Targets, with a Particular Focus on Ascending Aorta Aneurysms

**DOI:** 10.3390/antiox11020182

**Published:** 2022-01-18

**Authors:** Calogera Pisano, Umberto Benedetto, Giovanni Ruvolo, Carmela Rita Balistreri

**Affiliations:** 1 Department of Cardiac Surgery, Tor Vergata University Hospital, 00133 Rome, Italy; lindapisano82@gmail.com (C.P.); giovanni.ruvolo@unima2.it (G.R.); 2 Bristol Heart Institute, University of Bristol, Bristol BS2 8HW, UK; umberto.benedetto@bristol.ac.uk; 3 Department of Biomedicine, Neuroscience and Advanced Diagnostics (Bi.N.D.), University of Palermo, 90134 Palermo, Italy

**Keywords:** aorta diseases, ascending aorta aneurysm (AsAA), reactive oxygen species (ROS), oxidative stress, potential biomarkers, benefits and limitations, potential treatments

## Abstract

Aorta diseases, such as ascending aorta aneurysm (AsAA), are complex pathologies, currently defined as inflammatory diseases with a strong genetic susceptibility. They are difficult to manage, being insidious and silent pathologies whose diagnosis is based only on imaging data. No diagnostic and prognostic biomarkers or markers of outcome have been known until now. Thus, their identification is imperative. Certainly, a deep understanding of the mechanisms and pathways involved in their pathogenesis might help in such research. Recently, the key role of oxidative stress (OS) on the pathophysiology of aorta disease has emerged. Here, we describe and discuss these aspects by revealing some OS pathways as potential biomarkers, their underlying limitations, and potential solutions and approaches, as well as some potential treatments.

## 1. Introduction

In Western countries, the incidence of cardiovascular disease (CVD) is growing greatly because of the aging population. It is estimated that in 2030, the percentage of incidence will achieve the value of about 40.5% [1]. Thus, CVDs constitute a strong challenge to public health [2]. However, progress has been achieved in the management of some CVDs. For example, new thrombolysis and percutaneous coronary intervention procedures have been developed for the treatment of ischemic heart diseases. Such treatments have been able to improve both the quality of life and survival of the affected patients [3]. Conversely, for other CVDs, such as aorta pathologies (i.e., aneurysms, particularly sporadic ascending aorta aneurysms (AsAA) [4,5]), the management and outcome remain difficult. Consequently, the search for appropriate molecules and pathways with diverse applications from risk prediction and screening to diagnosis and prognosis, and the creation of specific algorithms that would be useful in preclinical and clinical settings, are imperative and encouraged [6].

In line with these observations, the identification of all the pathological conditions associated with aorta pathologies (endothelial dysfunction, medial degeneration, arterial hypertension, atherosclerosis, cardiovascular remodelling, senescence, death, etc.), their clinical typical features, and the mechanisms and pathways associated with their onset and progression have become the objects of intense research aiming to detect molecules which can be applied as potential biomarkers and targets for personalized treatments. Accordingly, biomarkers may be derived from clinical features, such as blood, ref. [7] fluid [8] or tissue investigations (for several “-omics”, e.g., genomics, transcriptomics, proteomics, metabolomics, microbiomics, etc.); clinical outcome scales; imaging data; physiologic testing; histological analyses and biochemical studies, just to mention a few. As mentioned above, biomarkers can facilitate diagnoses, predict the prognosis and clinical outcomes, and identify new therapeutic targets [9]. Another crucial use of biomarkers might also be accurately assessing the nature (beneficial, futile, or dangerous) of the biological effects of potential treatments. Accordingly, biomarkers, derived from imaging 3D data analysis [10] could precisely predict the risk of aneurismal rupture. Ideal biomarkers in aorta pathologies, particularly in AsAA, would allow us to identify the diverse stages of the diseases, and consequently distinguish between patients with or without the disease or clinical outcome of interest, such as dissection or rupture, with high sensitivity and specificity. Furthermore, they would be cost-efficient, rapidly obtainable without interfering with the administration of drug therapies, and non- or minimally invasive, as well as being widely significantly applicable in a universal manner via thoroughly standardized and realistically plausible methods and methodologies, even in settings with limited resources, without being influenced by confounding variables, including gender, age, ethnic background, diet, medications, circadian rhythm, environmental exposures, and other medical comorbidities [11,12].

Currently, it has emerged that the optimal field of research, in which aorta disease biomarkers can be easily detected, is oxidative stress (OS) and the consequent endothelial dysfunction [13]. Accordingly, it has been demonstrated that OS plays a fundamental role in the pathogenesis of all these diseases, AsAA included [13].

Based on these premises, we first describe the role of reactive oxygen species (ROS) and oxidative stress within the pathogenesis of aorta disorders, particularly in the onset of sporadic AsAA. Second, we show evidence how OS pathways and molecules add other important pieces to the intricate puzzle of the pathophysiology of aorta diseases, particularly in sporadic AsAA disease. This may allow the application of these molecules and pathways as potential biomarkers and targets in the management of these diseases, particularly in the complicated management of AsAA, whose diagnosis is currently based only on imaging evaluations and not on blood biomarkers; moreover, its treatment is exclusively by surgery (see the description in the next paragraph).

## 2. The Sporadic AsAA and Challenges in Biomarker Identification and Utilization 

Aneurysm of the ascending aorta (AsAA) is defined by the dilation of the ascending aorta’s diameter to greater than 1.5 times the normal diameter. Diverse forms of AsAA exist, including syndromic, non-syndromic and sporadic forms. Among these, the sporadic AsAA form is becoming a serious health risk factor in older Western people. Currently, it is estimated that aged individuals have an increased incidence of sporadic AsAA with advancing years. Epidemiological studies executed in geographic regions with stable populations with little outward or inward migration, such as in Minnesota and Sweden, demonstrate this evidence [14,15]. In addition, another determining factor related to the aging population is the increased number of hypertensive individuals [16]. Hypertension is a commonly prevalent and important risk factor for sporadic ASAA, as established by recent guidelines. Sporadic AsAA is a silent disease, until rupture or dissection occur, and is insidious in its onset and progression. The diagnosis of AsAA is often fortuitous and may occur during a routine physical examination or an independent medical evaluation. Once suspected, its identification is confirmed by clinical imaging modalities (e.g., X-ray, magnetic resonance imaging, computed tomography scanning, or ultrasound), which permit the selection of the appropriate surgical procedures (including elective surgery or endovascular repair), before the onset of catastrophic and life-threatening complications (i.e., dissection or rupture). Furthermore, until now, there have been no available biomarkers for early diagnosis of sporadic AsAA. On the other hand, sporadic AsAA has been the object of a very small number of investigations other than those into familial forms of the disease. As a result, it is difficult to generalize regarding the disease pathways or genetic risk factors that contribute to sporadic AsAA. In fact, this pathology of this disease has unclear mechanisms, and it has a very complex clinical presentation, characterized by the lack of overt symptoms until dissection or rupture occur. However, we and other groups have recently suggested that AsAA is an immune disease with a strong genetic component. Accordingly, the involvement of chronic innate inflammation is emerging, as well as the key role of oxidative stress (OS). The triggers of inflammatory responses, as well as the optimal mediators (e.g., in the respiratory burst of professional phagocytes) [17] include high levels of ROS, which produce OS [18]. Recent evidence has reported that ROS-induced OS is strongly linked to remodelling and degeneration of the aorta wall and especially of the aortic media [18]. This results in the development of important pathological conditions, including apoptosis of the smooth muscle cells, the fragmentation of elastic fibres, the degradation of collagen fibres and the infiltration of inflammatory cells. Furthermore, excessive ROS levels have been demonstrated to induce the release of matrix metalloproteinases (MMP) and stimulate the apoptosis of aortic smooth muscle cells (SMCs) [14,15,16,17,18,19,20].

Thus, AsAA has a very complex pathology, and therefore, one biomarker is not adequate for facilitating its management. Several pathways and mechanisms are involved in its pathogenesis. Therefore, this consideration leads to a paradigm shift away from the hunt for a single biomarker that can direct clinical care and research towards research into multi-biomarker profiles, which may include pro-inflammatory and OS pathways. 

## 3. OS in the Aorta Wall: Mechanisms and Pathways Involved in and Significantly Associated with Onset of Aorta Diseases, including AsAA

### 3.1. Physiological Actions of ROS in the Aorta Wall and the Stimulation of OS, with Immediate Effects on Cellular Components

**Figure 1:**
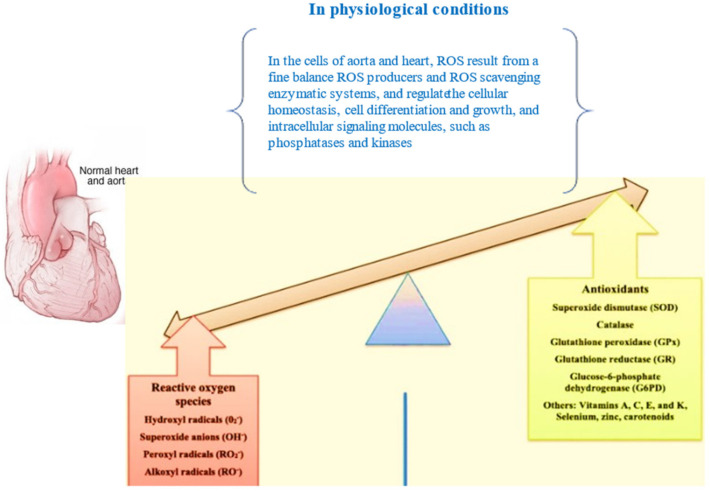
ROS in the aorta in physiological conditions: they regulate cellular homeostasis, cell differentiation and growth, and intracellular signalling molecules, such as phosphatases and kinases.

**Figure 2:**
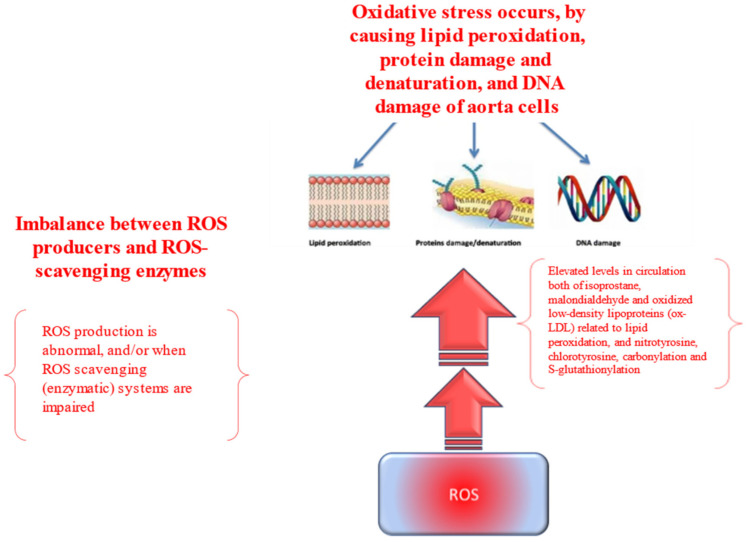
OS in the aorta wall: when ROS production is abnormal, and/or when ROS-scavenging (enzymatic) systems are impaired, OS occurs and causes cell damage or death because of lipid peroxidation of the biofilms of organoid and cell membranes, denaturation of proteins, decrease activity of several enzymes, DNA breakage and the consequent chromosome aberration. Elevated levels of isoprostane, malondialdehyde and oxidized low-density lipoproteins (ox-LDL) related to lipid peroxidation, nitrotyrosine, chlorotyrosine, carbonylation and S-glutathionylation have been assessed in patients affected by aorta disorders, such as aorta dilation and dissection.

**Figure 3:**
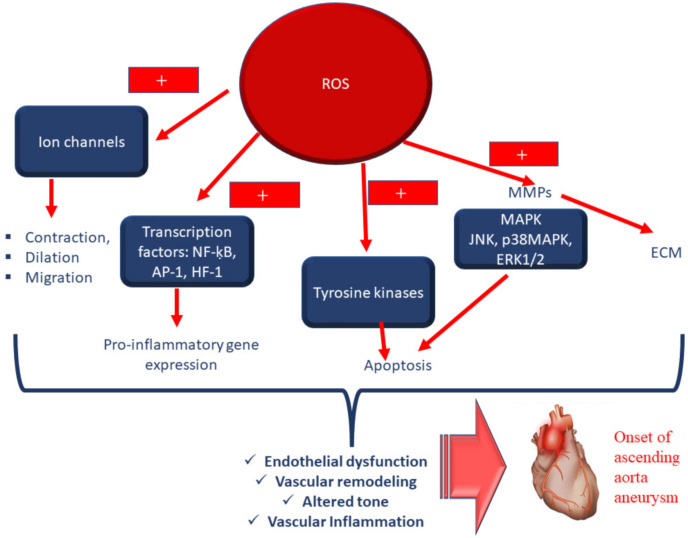
In conditions of OS (i.e., elevated levels of ROS), it has been demonstrated that ROS induce endothelium dysfunction, the release of matrix metalloproteinases (MMP) and apoptosis of aortic SMCs. Indeed, ROS modify the activity of tyrosine kinases, such as Src, Ras, JAK2, Pyk2, PI3K and EGFR, as well as mitogen-activated protein kinases (MAPK), particularly p38MAPK, JNK and ERK1/2. ROS may inhibit protein tyrosine phosphatase activity, (see Table 1), and activate NF-KB, AP-1 and hypoxia-inducible factor-1 (HIF-1), by evocating the release of inflammatory mediators. ROS stimulate ion channels, such as plasma membrane Ca^2+^ and K^+^ channels, leading to changes in cation concentration. Activation of these redox-sensitive pathways result in the media degeneration, altered vascular tone, and aorta wall remodelling, which represent the typical pathological conditions significantly associated with an increase in the dimensions of the aorta wall (i.e., with the onset of aneurysm) and dissection. In addition, ROS determine the increased release of inflammatory mediators, followed by the stimulation of inflammatory responses and a strong immune infiltration responsible for vascular inflammation. +, stimulatory effect; ECM, extracellular matrix; MMPs, matrix metalloproteinases.

ROS have a relevant physiological role in aorta cells, and their levels result from a fine balance between ROS producers and ROS-scavenging enzymatic systems [21,22,23] (see Table 1). Accordingly, ROS regulate cellular homeostasis, cell differentiation and growth, and intracellular signalling molecules, such as phosphatases and kinases [24,25,26,27,28] (see Figure 1). When this balance is absent, such as when ROS production is abnormal and/or when ROS scavenging (enzymatic) systems are impaired, OS occurs. OS causes irreversible cell damage or death caused by increased lipid peroxidation of the biofilms of organoid and cell membranes, augmented intracellular calcium levels, denaturation of proteins, decreased activity of several enzymes, breakage of DNA and the consequent chromosome aberration, and the activation of inflammatory responses, accompanied by the release of the related mediators [24,25,26,27,28] (see Figure 2 and Figure 3). Accordingly, elevated levels of isoprostane, malondialdehyde and oxidized low-density lipoproteins (ox-LDL) related to lipid peroxidation, nitrotyrosine, chlorotyrosine, carbonylation and S-glutathionylation have been assessed in patients affected by aorta disorders, such as aorta dilation and dissection [29,30,31,32,33,34,35]. Likewise, elevated levels of oxidative stress have been also detected in aortic tissues in animal models, and in cases affected by Marfan and bicuspid aortic valve syndromes, and AsAA [29,30,31,32,33,34,35].

### 3.2. OS and Aorta Media Degeneration and Remodelling: Associated Pathways 

Elevated levels of ROS [29,30,31,32,33,34,35] have also been demonstrated to induce both the release of matrix metalloproteinases (MMP, and the stimulation of apoptosis in aortic SMCs). These result in media degeneration and aorta wall remodelling, which represent the typical pathological conditions significantly associated with an increase in the dimensions of this aorta wall (i.e., with the onset of aneurysm) and dissection [36] (see Figure 3).

Angiotensin (Ang) II has been demonstrated to be involved in the aorta’s release of MMPs under OS conditions (see Figure 3). On the other hand, Ang II perfusion represents the optimal strategy for creating AsAA mouse models. Ang II stimulates the production of ROS in the aorta and inflammatory cells, promoting the mechanisms associated with the formation of aortic dissection or aneurysm [37], i.e., the release of MMPs [36]. Accordingly, it has been demonstrated in mouse models that a reduction of or, better still, a deficiency in nicotinamide adenine dinucleotide phosphate (NADPH) oxidase 1 (NOX1) significantly reduces the incidence of aortic dissection induced by Ang II perfusion via a significant decrease in MMPs [36]. In addition, it has been also reported that the treatment with ursodeoxycholic acid prevents Ang II-induced NOX1 expression and promotes the inhibition of apoptosis of SMCs [36,38] (see Figure 2). Similar results have been obtained by using human brain vascular SMCs stimulated with recombinant secreted proteins, acidic and rich in cysteine (SPARC) in vitro [39]. The observed results have revealed that SPARC proteins induce the increased expression of NOX proteins, especially NOX4, via the TGF-β1-dependent signalling pathway. This causes OS, pro-inflammatory matrix behaviour and apoptosis in human brain vascular SMCs. Thus, such proteins have been suggested to be involved in the onset of intracranial aneurysms by stimulating OS and the release of MMPs via activation of the TGF-β1-dependent signalling pathway [39]. Accordingly, the TGF-β1/ROS/NF-κB pathway has been demonstrated to mediate these effects, and vascular SMC senescence and aneurysm formation in AsAA, BAV and Marfan syndrome patients [40,41,42,43,44,45]. Consistent with this, increasing evidence has reported that OS can activate the NF-κB pathway (see Figure 3), which is a well-recognized inflammatory driver of AsAA pathogenesis. This promotes the release of cytokines, which contribute to further recruitment of circulating monocytes to the middle aorta and their differentiation into active macrophages that can secrete MMPs and other ECM-degrading proteins and to consequently accelerate the onset and progression of AsAA [40,41,42,43,44,45] (see Figure 3). 

In vitro and ex vivo studies on human aorta tissues have also shown that ROS accumulation is significantly associated with the increased expression of the connective tissue growth factor (CTGF), whose levels appear to correlate with media degeneration [46]. Accordingly, CTGF has been demonstrated to regulate the synthetic phenotype of vascular SMC. This finding has been validated by using a murine model of AsAA (C57BL/6J) based on Ang II infusion. The findings revealed that medial thickening and luminal expansion of the proximal aorta are associated with the vascular SMC synthetic phenotype as observed in human aorta tissue samples [46].

Moreover, it has been also demonstrated that NOX, xanthine oxidase (XO), myeloperoxidase, (MPO), lipoxygenase (LOX), cyclooxygenase (COX), uncoupled endothelial nitric oxide synthase (eNOS), other amine oxidases and non-enzymic sources, including electron leakage from the mitochondrial electron transport chain, result in altered expression and function in patients with AsAA (see Table 2) [47].

### 3.3. Molecules and Pathways Related to OS Attenuation in Aorta Wall 

Sirtuin3 (Sirt3), a histone deacetylase with the function of regulating many cellular processes, has been demonstrated to reduce ROS levels, inflammation, and the apoptosis of aorta SMCs. Sirt3 deficiency has been investigated and reported to be significantly related to enhanced OS. Mice with thoracic aorta dissection have been shown to have a significantly decreased expression of Sirt3 compared with normal mice. Mice with Sirt3 knockout displayed a significantly increased incidence of thoracic aorta dissection and aorta dilatation. In addition, an increase in Sirt3 overexpression has been associated with a significant reduction in Ang II-induced ROS production, NF-kB activation, and apoptosis in human aortic SMCs. Consequently, Sirt3 overexpression diminishes aneurysm formation and reduces aortic expansion. Thus, Sirt3 deficiency is significantly associated with an increased susceptibility to of thoracic aorta dilation and dissection, because the Sirt-3-induced anti-ROS effects are attenuated versus a significant increase in apoptosis of aorta SMCs and inflammation [48]. 

In contrast to CTGF activity and the mentioned enzymes above, and similarly to the actions mediated by Sirt3 [41], diverse intracellular enzymes, including superoxide dismutases, catalase, glutathione peroxidases and peroxiredoxins (Prdxs) (see Figure 1 and Table 2), have the role of maintaining precisely balanced ROS levels to reduce their eventual increase during the pathogenesis of diseases such as AsAA [49]. However, evidence has recently reported that the impaired function of these enzymes, as well as mitochondrial dysfunction, characterize patients affected by AsAA, as elegantly summarized by Portelli and co-workers [47]. Particular attention has been also given to Prdxs, antioxidant enzymes involved in the regulation of OS and H_2_O_2_-mediated intracellular signalling by other research groups. Accordingly, studies have confirmed the impaired activity and expression of Prdxs, particularly of Prdx2, in patients affected by AsAA [49]. 

## 4. Suggestions and Recommendations for Further Investigations into the Ideal Scenario for Biomarker Profile Development 

The selection and development of biomarkers should be based on a clear understanding of the pathogenesis of aorta diseases, particularly of AsAA (here taken as typical example), their subtypes (i.e., sporadic, syndromic, and non-syndromic forms, in the case of AsAA), relevant complications (i.e., dissection and rupture) and recovery phase. With that in mind, several of the OS pathways and molecules described above do not completely achieve this purpose. They can partly explain the close relationship between OS and the onset of aorta diseases, such as AsAA, but they do not allow us, for example, to reveal the symptoms of severity injury, which might help a practitioner to immediately intervene or predict complications. In addition, given the long-term functional impairment of the aorta wall caused by OS and the related inflammation, identifying diagnostic biomarkers that correlate with the severity of injury and prognostic biomarkers for the early prediction of dissection and rupture, as well as diagnostic biomarkers for monitoring the increase in aorta dilation, the peak and the resolution with surgery or non-invasive treatments, might serve in the care and critical management of AsAA patients. Furthermore, understanding the dynamic and temporal biomarker levels relative to the patient’s clinical course would shed important light on the pathophysiology of AsAA and reveal novel putative therapeutic targets. In terms of biosamples, blood samples would be preferable because of their ease of collection and rapid examination. It would additionally be ideal to collect blood samples at precise time intervals to obtain temporal profiles, which might also be correlated with initial clinical imaging severity. Comparing the rates of change would delineate the contribution of tissue (aorta) versus systemic mechanisms that are fundamental to developing effective biomarkers and therapeutic treatments. 

However, all these observations suggest that further scientific efforts are needed for the identification of biomarkers related to OS for aorta pathologies, i.e., AsAA, and of targets for the development of antioxidant therapies. To this end, further advances could be realized by studying further molecules associated with entire OS pathways through a new technological appraisal based on innovative approaches and systems. The integration of multi-omics analyses based on genomics, epigenomics, transcriptomics and proteomic profiles, along with recent metabolomic, microbiomic and nutrigenomic investigations should be encouraged [50,51,52]. This could attenuate the limitations regarding the current use of the many available methods for detecting ROS and ROS damage, as recently suggested in the recommendations of the American Heart Association [53]. In addition, this type of analysis would bring together clinicians and basic/translational researchers into aorta diseases, such as AsAA, for the intriguing opportunity of reverse-translating the biomarkers. Likewise, this method might also be used to develop pharmacological or non-pharmacological translational treatments. In the next section, we describe and discuss some of these treatments.

## 5. From the Experimental Aspects to Translational Medicine: Antioxidant Treatments and Targets

Several pharmacological treatments and natural compounds with antioxidant properties have been developed. Below, we describe some of these, showing their beneficial effects and limitations.

### 5.1. Natural Compounds and the Mediterranean Diet

Natural compounds, principally obtained from plants but also from animals and micro-organisms, have been widely recognized for thousands of years as treatments for the prevention of and therapy for many human diseases [54,55]. Of note are the phytochemicals, chemical compounds produced by plants for resisting pathogens, which have beneficial properties for human health. Accordingly, they have biological effects on diverse mechanisms, such as epigenetic modifications, modulation of signal transduction and metabolic pathways, and regulation of antioxidant enzyme activity [54,55]. In support of their advantageous effects, several data have been reported in literature, particularly regarding their anticancer activities [56] and their ability to delay or escape the onset and progression of CVD [57] or other chronic pathologies [58]. The phytochemicals with antioxidant-rich properties comprise the polyphenols, flavonoids, iso-flavonoids, anthocyanidins, phytoestrogens, terpenoids, carotenoids, limonoids, phytosterols, glucosinolates and fibres. They are generally utilized as functional foods, soft drinks, and many other food items, and show good nutritional value [59,60]. In animal AsAA models, their action has been revealed to be effective, while human clinical trial studies have failed to demonstrate this evidence [61,62,63,64,65,66,67]. Davies and Holt, by adopting two modelling techniques, namely Gillespie’s Stochastic Simulation Algorithm (using Kinetiscope) and a discrete Markov chain, have showed the diverse reasons for this failure, including the rate of reaction between the free radicals and the non-enzymatic antioxidants, which is considered a necessary threshold for evaluating the effect of an antioxidant on the free radical defence systems naturally present, making it able to minimise their action [63]. In addition, it has been also reported many times that an active natural compound may prove to be inactive because of the isolation method [63]. Accordingly, the development of recent analytical and computational techniques offers new possibilities for treating and processing complex natural compounds for obtaining new and innovative drugs [64]. The use of quantum computing, computational software and databases allow the simulation of molecular connections and calculation of the characteristics and factors required for the development of drugs, even if they are natural, and these tools can also be used for evaluating their pharmacokinetic and pharmacodynamics, helping us reach this difficult goal, as recently described by Thomford and co-workers [64].

Despite these limitations, new flavonoids have been tested, including diosmetin. Diosmetin is a citrus flavonoid with antioxidant and anti-inflammatory effects. Experimental studies have shown its ability to ameliorate vascular dysfunction and remodelling by influencing the expression of Nrf2/HO-1 and p-JNK/p-NF-κB in hypertensive rats [65]. Other natural compounds explored in recent examinations include, for example, essential oils, which represent alternatives to natural and synthetic antioxidant agents [66,67]. Among these, the essential oil of *Inula montana*, containing the E,E-farnesyl acetate was shown to be a new inhibitor with potent activity towards superoxide dismutase (SOD) and ctDNA inhibition [68]. Another area of recent interest is hydroxytyrosol (HT), the major phenolic compound in olive oil. HT has beneficial properties, such as its remarkable antioxidant and anti-inflammatory power [69]. Accordingly, recent studies have considered the role of HT in the formation of advanced glycation end-products (AGEs), which are associated with the onset of diabetes, and neurodegenerative and cardiovascular diseases. The data obtained have demonstrated the capacity of HT to selectively inhibit the protein glycation reaction in human insulin and to counteract the AGE-induced cytotoxicity by acting on sirtuin levels and oxidative stress, as well as on the inflammatory response [69].

Consistent with these recent discoveries about HT, it has also been described that the Mediterranean diet can positively impact the cardiovascular system thanks to its antioxidant effects [70]. Precisely, two studies have tested the risk of abdominal aortic and cerebral aneurysms in relation to Mediterranean diet adherence [71,72]. They have been conducted on a very large sample and have demonstrated that a Mediterranean diet based on a high consumption of fruits, vegetables, wholegrains, legumes, nuts, fermented dairy products, fish, and olive and/or rapeseed oil; moderate consumption of alcohol; and low consumption of processed and unprocessed red meat may represent a positive factor for the prevention of two forms of aneurysm [71,72]. There have been no studies about AsAA and the Mediterranean diet to date. However, the positive results described here may encourage us to conduct this type of study. On the other hand, preclinical studies, population studies and clinical trials recommend adherence to the MD particularly the consumption of foods with a high content of polyphenols, such as biophenols, including red wine, extra virgin olive oil (EVOO), green tea, spices, berries, and aromatic herbs [73,74]. Although biophenols are present in low quantities in these foods, their quotidian consumption during the life of an individual may result in a significant reduction in the incidence of aorta diseases, such as AsAA [73,74].

Of note also are the recent findings on quercetin, a natural flavonoid, commonly existing in nature, especially in tea, coffee, apples and onions [75]. Specifically, some very promising studies have shown how the combination of quercetin with small antioxidant agents, such as resveratrol, luteolin, arctigenin, trehalose, curcumin, etc., can improve the therapeutic effects at lower doses by preventing possible toxicity and its subsequent effects during treatment [75]. Likewise, it has been recently observed that the effect of other antioxidant agents may be increased by fusing or combining two or more compounds with different properties [76]. One example was given by a recent study, which tested the effect of non-natural compounds of non-steroidal anti-inflammatory drugs fused with the antioxidant fractions 3,5-di-tert-butyl-4-hydroxybenzoic acid (BHB), its reduced alcohol 3,5-di-tert-butyl-4-hydroxybenzyl alcohol (BHBA), or 6-hydroxy-2,5,7,8-tetramethylchromane-2-carboxylic acid (Trolox), a hydrophilic analogue of α-tocopherol [76]. For validating the anti-inflammatory and antioxidant effects of these compounds, machine learning algorithms have been used. Fortunately, interesting results have been obtained, which have shown the significantly increased antioxidant and anti-inflammatory activities of the new fused molecules compared with the parent molecules [76].

Thus, the combination and design of multifunctional compounds might potentially be more advantageous and could represent a more efficient therapeutic approach to diseases such as AsAA, as discussed in this article.

### 5.2. Innovative Technologies as Targeted Antioxidant Treatments: Nanomedicine and Its Benefits and Limitations

Modern technologies have been recently developed for treating OS and preventing the onset of aorta diseases, such as AsAA. Among these, the use of nanomaterials and nanotechnologies [77,78] allows us to apply nanomedicine, specific targeted treatments that are able to improve the delivery of targeted drugs (e.g., natural antioxidant compounds or pharmacological drugs) using nanoparticles (NPs) (e.g., liposomes and niosomes plus polymers, lipid and organic polymer hybrids and precursors, carbon nanotubes, quantum dots, metal, metal oxides), and to investigate their bioavailability, as well as to reduce the associated toxicity or side effects, and costs. Such innovative approaches have been used, for example, for increasing the bioavailability, stability, and the consequent beneficial effects of curcumin against inflammatory-related diseases [79]. Likewise, pH and ROS dual-responsive NPs engineered by integrating pH- and ROS-responsive cyclodextrin materials with resveratrol have been used as an efficient and secure nanoplatform for therapeutic delivery to the sites of vascular inflammation, identified by the presence of acidosis and OS [80,81]. These promising results suggest the use of this approach for ameliorating ROS levels and reducing the susceptibility to aorta diseases, such as AsAA.

However, in the application of nanomedicine, some issues regarding about its benefits would need to be explored [78,79]. Specifically, nanomedicine based on use of nanoparticles encapsulated with therapeutic compounds offers the advantage of overcoming the body’s biological barriers and improving the method of delivering compounds to specific tissues and organs with high levels of ROS, such as the aorta, in our specific case. Furthermore, nanomedicine technology has another advantage, namely its ability to enhance the efficacy of therapeutic compounds by reducing their toxicity or other side effects. Nanoparticles constitute one of the major technologies of nanomedicine, because a combination of physical, chemical, and biological technologies may be used for improving the in vivo performance of this next-generation therapeutic approach [78,79]. Another important aspect of nanomedicine is assessment of the biodistribution of the nanoparticles following their in vivo administration in animals and humans [78,79]. This constitutes a challenge despite the large range of techniques available for detecting nanoparticles’ biodistribution, including histology, electron microscopy, liquid scintillation counting (LSC), indirectly measuring drug concentrations, in vivo optical imaging, computed tomography (CT), magnetic resonance imaging (MRI) and nuclear medicine imaging. Despite the diverse benefits, which encourage their use, additional investigations are certainly needed for making this approach very effective [82].

### 5.3. New Drugs: Metformin and Melatonin, and the Necessity of Validating Positive Evidence

For stopping or retarding the development of aorta diseases, such as AsAA, and reversing its progression into aortic dissection, potential pharmacological treatments currently under investigation include β-adrenergic blocking agents, losartan, irbesartan, angiotensin-converting-enzyme inhibitors, statins, antiplatelet agents, and doxycycline, which represent the elective treatments used for AsAA [83,84]. However, as mentioned above, other drugs, such as those with antioxidant action, are the object of several studies. Among these, metformin, a drug usually used in diabetes therapy, is emerging as a potent antioxidant agent [85]. Studies on model organisms have demonstrated that metformin can ameliorate the OS status and health status of the cardiovascular system, including the aorta, via diverse mechanisms: (a) reducing insulin and IGF-1 signalling, (b) inhibiting mTOR, (c) reducing the levels of ROS, (d) lowering inflammation, (e) reducing DNA damage and (f) activating the AMPK/acetyl-CoA carboxylase (ACC) pathway [85]. The effect on the AMPK/acetyl-CoA carboxylase (ACC) pathway has attracted particular attention [85]. Consistent with this, Li and co-workers have recently demonstrated that metformin inhibits the onset of intracranial aneurysm and its progression by controlling vascular smooth muscle cell phenotype switching via the AMPK/ACC pathway [86]. In addition to the recent work by Li’s group, other studies have suggested that metformin can suppress the progression of early aneurysms, such as abdominal aortic aneurysms [87]. This aspect has been recently pointed by Yu’s group, who conducted a systematic review and metanalysis of eight studies and 29,587 participants [87]. It appears to be relevant that in every population examined in these eight studies, a significant inhibitory effect on aneurysm growth in the cases prescribed metformin for Type 2 diabetes management was demonstrated, with a variation only in the magnitude [87]. Another interesting aspect reported by Yu’s group concerns the few results existing in the literature on the efficiency of metformin in controlling the onset of aneurysm in non-diabetic patients, even if various clinical trials have examined novel endpoints in other cardiovascular disorders, cancer and other pathologies related to OS status [88,89]. Thus, this aspect needs to be clarified, although clinical trials on metformin with this objective are also planned or ongoing in European and American populations [89].

In the literature, the existing data allow us to suggest that metformin currently represents the most likely and efficient candidate as an antioxidant and protective agent against all the forms of aneurysm, even if insignificant or even no data exist on AsAA. This question should be definitively addressed by performing an adequate number of clinical trials.

Another emerging antioxidant molecule is melatonin (N-acetyl-5-methoxytryptamine), an indoleamine molecule highly and generally identified in many plant and animal organisms, including humans [90,91]. Melatonin is synthesized from the essential amino acid l-tryptophan due to the action of four enzymes. In vertebrates, including humans, it is known as a secretory product of the pineal gland, even if it is a physiological cell component of other tissues, such as the retina, skin, immune system, gastrointestinal tract, and reproductive tract [91]. In these, melatonin is present at diverse levels, with a higher density within the membranes and the mitochondria, where it has several functions, including interacting with lipids, stabilizing all cellular membranes, reducing lipid peroxidation, and increasing ATP production. In addition, it produces several beneficial effects in the context of antioxidant activities. Specifically, it has antioxidant and free radical scavenging capacity against ROS and reactive nitrogen species (RNS), which helps melatonin to protect proteins and mt-DNA from OS [91]. In addition, it increases the activity of endogenous antioxidant enzymes and has anti-inflammatory properties related principally to SIRT1 activation [92]. Accordingly, melatonin has displayed, in apolipoprotein E-deficient mice, the ability to reduce endothelial damage, the loss of SIRT1 and endothelial nitric oxide synthase, and the expression of p53 and endothelin-1. In addition, it has also been noted that melatonin confers a cardioprotective effect against myocardial ischemia reperfusion injury by reducing oxidative stress damage via activation of SIRT1 signalling in a receptor-dependent manner [92]. Likewise, it has been recently demonstrated in thoracic aortic aneurysm and dissection (TAAD) mouse models that melatonin shows therapeutic effects against TAAD by reducing OS and VSMC loss via activation of SIRT1 signalling in a receptor-dependent manner, thus revealing a novel therapeutic strategy for TAAD. Of course, basic experimental investigations and clinical trials are necessary for its future clinical applications in AsAA [93].

## 6. Prebiotics, Probiotics and Synbiotics as Other Therapeutic Possibilities for OS and Aneurysm

The gut microbiota is described as having a substantial influence on the health of an individual, and alterations in this, termed dysbiosis, play a key role in the in pathogenesis of several CVDs, such as aneurysms, contributing to systemic endotoxemia and inflammation, atherosclerosis, and hypertension [94,95,96,97]. Accordingly, a recent study conducted in C57BL ApoE^−/−^ mice with abdominal aortic aneurysms, induced by Ang II (1000 ng/min per kg), demonstrated that the composition of the gut microbiomes was diverse between control and AAA mice and was correlated with abdominal aortic aneurysm diameter. Moreover, linear discriminant analysis showed that the effect size of the genera *Akkermansia*, *Odoribacter*, *Helicobacter* and *Ruminococcus* correlated with the progression of abdominal aortic aneurysm [98]. Thus, gut microbial dysbiosis might contribute to the pathogenesis and progression of abdominal aortic aneurysm and represent a potential target for further research [98]. Recent evidence also suggests that the origin of aorta diseases, such as aneurysm, may derive from early life conditions. Accordingly, an increased number of studies have reported that the health status of the gut microbiota in early life is significantly associated with the onset of aorta diseases in later life. In turn, a growing body of evidence has noted the close connection among the mitochondria, the gut microbiome and ROS [94,95,96,97,98]. An imbalance in the gut microbiome leads to mitochondrial dysfunction and elevated levels of ROS. These reciprocally impact human health, the homeostasis of gut cells and the gastrointestinal microbial community’s biodiversity [94,95,96,97,98]. All this evidence has led research to delve into gut microbiota-based treatment modalities, including probiotics, prebiotics and synbiotics [99], which can restore symbiosis. Probiotics are represented by beneficial living microorganisms, which can improve the intestinal microbiota profile. Prebiotics comprise non-digestible substrates that can increase the growth of beneficial gut microorganisms, such as the resident microorganisms and probiotic strains. Combinations of probiotics and prebiotics are known as synbiotics, which can synergistically impact the gastrointestinal tract [100,101,102,103,104]. Experimental and clinical studies have reporting promising results [105]. Specifically, it has been shown that probiotics reduce cholesterol levels, and increase bile salt synthesis and bile acid deconjugation. Comparable effects have also been detected for prebiotics and synbiotics [105]. However, probiotics also seem to have anti-oxidative, anti-platelet and anti-inflammatory properties. In addition, probiotics show beneficial effects in all the studied models in vitro, in animal models and in humans, where probiotic supplementation diminished the OS and aorta diseases risk [105]. However, the commercially produced probiotics, prebiotics and synbiotics have properties which remain unidentified; consequently, additional experimental investigation is required for evaluating their ability to prevent and treat OS. Well-designed clinical trials are particularly mandatory for assessing the impact of probiotics on trimethylamine-N-oxide (TMAO), which is supposed to be a cardiovascular marker, and to elucidate the long-term effects and action of probiotic, prebiotic and synbiotic supplementation in combination with other drug therapies (for example, aspirin). Others are evaluating if probiotics can upregulate altered genes in aorta diseases, such as aneurysms, and promising data have been obtained [106].

However, while it cannot be unambiguously indicated whether such supplementation generates benefits for the prevention and treatment of aorta diseases, it is of note that clinical studies performed up to the present have revealed their beneficial effect on OS and dysbiosis without any side effects to their use.

## 7. New Regulators of OS for Developing Targeted Treatments

As mentioned above, OS can modulate and alter the expression of gene profiles related to tissue homeostasis and the onset of associated diseases. Concerning the expression of genes, RNA-binding proteins (RBP) have a key role in RNA expression and metabolism. Consequently, the appropriate control of these proteins is critical for cellular health [107]. Use of a clustered regularly interspaced short palindromic repeats (CRISPR)-Cas9-single guide RNA library and stimulation of cells with paraquat has permitted us to identify CSDE1 and STRAP proteins, which interact with each other and produce sensitivity to OS, and the Pumilio homologues (PUM1 and PUM2), which produce resistance [98]. Thus, this study emphasized that the use of genetic screening may allow us to identify RBPs and novel genes regulating the sensitivity to OS. They may be used for developing targeted treatments for OS [107].

Another class of novel OS regulators is the circular RNAs (circRNAs) [107,108], endogenous non-coding RNAs characterized by a covalently closed-loop structure generated through a special type of alternative splicing termed back-splicing. At this time, growing evidence has disclosed that circRNAs are evolutionarily conserved across species, stable and resistant to RNase R degradation. They display cell-specific and tissue-specific/developmental-stage-specific expression. Their biogenesis appears to be dissimilar to the canonical splicing of linear RNAs and is controlled by specific cis-acting elements and trans-acting factors [107,108,109,110,111]. CircRNAs act as regulators of diverse biological and pathological processes by sponging miRNAs and binding to RBP, which are, as mentioned above, regulators of splicing and transcription, modifiers of parental gene expression and regulators of protein translation, or by being translated into peptides in various diseases [107,108,109,110,111]. CircRNAs have been detected in exosomes and body fluids, including human blood, saliva, and cerebrospinal fluids, showing that these exo-circRNAs can represent both disease biomarkers and novel therapeutic targets. Specifically, exo-circRNAs can be used as biomarkers of OS because they are regulated by OS and induce ROS production and cause ROS-induced cellular death, cell apoptosis and inflammation. These features make circRNAs important regulators of diverse diseases, such as atherosclerosis and other diseases of the cardiovascular system, metabolic disease, and cancers [107,108,109,110,111].

Based on this concept, circRNAs might represent another effective therapeutic approach to OS. Controlling the expression of circRNAs in cardiovascular cells might permit the application of anti OS agents for significantly reducing the endothelial dysfunction and vascular inflammation associated with the onset of CVD (e.g., aneurysms) and metabolic diseases [107,108,109,110,111].

## 8. Conclusions and Perspectives

It appears evident that OS plays a role in the pathogenesis of different forms of aorta diseases, AsAA included, even if additional studies are required for clarifying the pathways involved. This might facilitate the identification of biomarkers. Here, we have reported some of these and suggested potential targets for developing related treatments. Of note are the RBP proteins and circRNAs, which are emerging not only as OS regulators but also as biomarkers and potential targets for treatment. In additional, our attention has been particularly given to emerging natural antioxidant agents, such as diosmetin, as well as combinations of these, e.g., quercetin with resveratrol, luteolin, arctigenin, trehalose, curcumin, etc. These have been demonstrated to have therapeutic effects at lower doses. However, studies have also shown their possible toxicity and its consequent effects during treatment [75] and demonstrated their failure to be beneficial for AsAA in human clinical trial studies because of some factors. Possible solutions have been suggested, as well as the use of more innovative technologies, such as NP or emerging drugs, i.e., metformin or melatonin, although further studies are needed to validate the beneficial evidence. Particular attention has been also given to the Mediterranean diet, as well as to gut microbiota-based treatment modalities, including probiotics, prebiotics and synbiotics [99,100,101,102], which can restore symbiosis, since a close link has been demonstrated between gut dysbiosis and AsAA.

Certainly, all this evidence indicates and clarifies some aspects of the complex link between OS and the onset and progression of aorta diseases, such as AsAA, as well as encouraging the treatments for stress, but it still appears incomplete. Many gaps and limitations characterize it, as reported above. Consequently, further investigations are needed, possibly of a multi-omics nature, which could provide a complete portrait with the translational aim of identifying biomarkers and innovative therapies to apply in the complex management of aorta diseases, such as AsAA, by facilitating diagnosis, which is currently based only on imaging evaluations, whereas treatment is exclusively founded on surgical approaches.

## Figures and Tables

**Table 1:** The most important enzymes and molecules involved in ROS production.

Enzymes and ROS Molecules	Biological Effects
Glutathione (GSH) system	Preventing the initiation of oxidative stress Endothelial and smooth muscle cell dysfunction
Metallothionein (MT)	Preventing the initiation of oxidative stress-induced aortic MMP-9 expression
Superoxide dismutases (SOD)	Transforming RNS/RNS into less reactive species
Glutathione peroxidases (GPx)	Transforming RNS/RNS into less reactive species
Peroxiredoxins (PRX)	Transforming RNS/RNS into less reactive species
Nox4	Oxidative damage to multiple cytoskeletal and contractile proteins, and elastic fragmentation
Xanthine oxidase (XO)	Alteration of contraction and relaxation, ECM degradation and aortic wall remodelling
SmgGDS (Small GTP-Binding Protein GDP Dissociation Stimulator)	Maintaining the contractile phenotype of VSMCs. A deficiency induces severe aortic dilatation and severe elastic fragmentation, higher levels of ROS and MMPs, and inflammatory cell migration
Myeloperoxidase (MPO)	Increased MMP-2 and MMP-9 expression; increased ECM fragmentation and apoptosis
ERK1/2 signalling pathway	The increased activation of non-canonical ERK1/2 signalling due to inflammatory cells is known to be associated with TAA pathogenesis, predominately through the downstream effect of increased MMP expression
Myeloperoxidase (MPO)/hypochlorous acid (HOCl) system	The MPO/HOCl system produced in neutrophils has been demonstrated to be a local mediator of tissue damage, particularly in ascending aorta aneurysms
Nox 2 and Nox 4	A specific increase in endothelial ROS production in Nox2 transgenic mice was sufficient to cause Ang II–mediated aortic dissection

**Table 2:** Molecules involved in oxidative stress.

Enzymes	Biological Effects
NADPH oxidases (NOX1, NOX2)	Generation of ROS Major sources of ROS in the artery wall The NOX2 phagocyte plays a role in catalysing the respiratory burst NOX2 facilitates oxygen reduction, resulting in the formation of superoxide
MPO (enzyme myeloperoxidase)	MPO is a peroxidase; excessive levels of these toxic molecules cause tissue damage. MPO-producing macrophages can infiltrate TAA and are likely involved in the progression of TAA disease. MPO can exacerbate mechanisms of damage to the ECM and DNA, activation of inflammatory signalling and an increase in endothelial dysfunction
ERK1/2	Increased MMP expression Increased oxidative stress due to inflammation Can disrupt aortic wall homeostasis by altering the characteristics of VSMCs Promotion of human aortic VSMC migration
3-Nitrotyrosine	Markers of MPO-mediated oxidative damage Promotion of human aortic VSMC migration Contributing to VSMC dysfunction
3-Chlorothyrosine	Markers of MPO-mediated oxidative damage Promotion of human aortic VSMC migration
MAPK signalling pathway	Expression of relevant target genes, including the upregulation of MMPs
MPO-derived oxidants	DNA modification, causing DNA damage Affecting VSMC DNA
MPO-derived HOCl	Damage to specific DNA bases Inhibition of DNA repair enzymes
Nitric oxide (NO)	Important regulator of vascular tone Reduced NO production causes endothelial dysfunction
NOX4	Reduced elastin fragmentation, less endothelial dysfunction and an increase in contractile markers
Glutathione peroxidase (GPx) and glutathione reductase (GR)	Higher lipid peroxide fluorochromes, expressed as U/g aorta, than in controls in both abdominal aortic aneurysms (AAA) and atherosclerotic occlusive disease (AOD)
